# Transactions of the Buffalo Obstetrical Society

**Published:** 1884-10

**Authors:** 


### Transactions of the Buffalo Obstetrical Society.

*Meeting August 26, 1884.*

The Vice-President, Dr. R. L. Banta, in the Chair.

Dr. W. W. Potter read a paper on " The Obstetrical Management of the Perinæum."

The paper embraced three propositions: First, is the perinæum vested with that importance, anatomically and physiologically, to warrant any special care during parturition? Second, if this is admitted, how shall we best prevent its rupture? Third, failing in this, what shall be done with reference to its immediate restoration?

The essayist took ground with the more modern opinions, in regard to the relations of a sound perinæum to the good health of woman, holding that it played an important part in maintaining the pelvic viscera in their normal position; that its office is, in part, to strengthen that part of the pelvic floor which has no bony support; and, finally, that the integrity of the female perinæum, and, to a great extent, the proper relations of the pelvic organs, depend upon a perfect perineal body.

For our improved anatomical knowledge of the part, acknowledgments were made to the researches and dissections of Henle, Savage, Ranney, Hart and Foster.

" The frequency with which lacerations of the perinæum occur is so differently related by observers, that anything like correct statistics are unobtainable. One class of practitioners, and I should presume by far the larger class, attaching very little importance to the lesion, considers only cases where the perineal body is severed from end to end; while another class, appreciating the value of a sound perinæum, reports the simpler breaches of continuity as perineal rupture. There are others, again, who never inspect the parts after delivery, and assert, with an air of grandiose superiority, that they 'never have lacerations of the perinæum in their practice.' "

" In this connection," says the essayist, " it would seem proper to remark, that in the very nature of things a certain number of lacerations are unavoidable, and the occurrence of the accident is no proof of unskilfulness on the part of the accoucheur. When it does occur, however, it is becoming, in the present light of science, that it should not escape observation, and that measures to restore it, immediate or remote, should not be neglected."

How shall we best prevent laceration of the perinæum when threatened in labor?

*First.* Never give ergot until the *completion* of labor. Great stress was laid upon this dictum, and the reasons therefor discussed at some length.

*Second.* The rectum must be thoroughly emptied prior to the commencement of the second stage.

*Third.* The patient must be made to assume the left-lateral posture during the passage of the fœtus through the vulvar orifice.

*Fourth.* As to support of the perinæum. Great diversity of opinion exists among obstetricians as to the propriety of rendering artificial support to the perinæum during the final passage of the child. Much has been said, pro and con, on this subject, and the question if any direct protection ought to be used or not may be regarded as still *sub judice.* Even great diversity of opinion exists among the advocates of "support," as to the best method of rendering it.

After passing in review the various methods recommended, the reader advocated but two, viz.:

*First.* The reinforcing of the vulvar ring with very moderate pressure of the flat of the hand, placed so that the fold between the thumb and index finger rests against the posterior commissure, while the presenting part is dilating the vulvar orifice.

*Second.* By direct pressure upon the presenting part of the fœtus, and, finally, when the vertex protrudes, to lay hold of the chin with two fingers of the left hand in the rectum, and thus control the delivery of the head. The head, in this way, can be gradually extended, kept away from the perinæum, pushed firmly against the pubes, and the pressure equalized against all parts of the vagina, to a considerable extent. The perinæum is carefully watched, different portions of the vulva are pushed backwards, slipped gently over the head, and the chin caused slowly to advance until finally it glides out of the vagina. Chloroform, administered simultaneously with the seizure of the chin, and maintained in its effects until the delivery of the head is accomplished, will enable us to assume entire control of delivery during the enucleating process.

The forceps, and episiotomy, were remarked upon as having very little place in the prevention of laceration of the perinæum, *serre fines* pronounced a delusion and a snare, and the paper closed with a plea for primary perineorrhaphy, and a brief description of the operation, the following being the concluding sentences:

"There are, no doubt, sources of failure inherent to the primary operation itself; for instance, in cases where there has been much delay, or much manipulation within the parturient canal, whereby a semi-pathological condition of the parts is produced; for in such, if the perinæum becomes torn, the reparative process is very much retarded, and union does not take place if it is sutured. Equally fruitful sources of failure are, however, to be found in the performance of the operation in a hurried or unskilful manner. With the improved methods at our command, and the constantly accumulating experience, the failures are being rapidly reduced to an insignificant minimum."

*Discussion*—Dr. Frederick, in opening the discussion, remarked that it was important to exhaust every useful method to preserve the perinæum intact. Its usefulness was hardly over-estimated, and he leaned to the side of regarding it of prime importance in the preservation of the normal relations of the pelvic organs. He did not think much good could come of perineal support, as ordinarily understood and as generally taught. The better plan is to retard the head by pressure, when it is distending the vulvar orifice and the perinæum is threatened. He had frequently performed episiotomy in hospital practice and the cases did well. In case of rupture he favored the treatment by immediate suture.

Dr. Stockton approved, in the main, the principles advanced by the essayist, but desired to enter his protest against the too sweeping condemnation of ergot, which seemed to be quite the fashion now-a-days. He is in the habit of giving the drug just before the termination of the second stage, and believes that it is useful in preventing post partum hemorrhage, as well as in promoting the expulsion of the placenta. He is also in the habit of treating perineal ruptures by immediate suture, and obtains good results.

Dr. Van Peyma has not been in the habit of repairing perineal rents primarily in his practice; has not found occasion to do so; thinks it a good plan to give ergot near the close of the second stage. In regard to supporting the perinæum with a view to prevent rupture, thinks very little good can come from any method yet proposed, but favors retarding the head by direct pressure, in some cases. He also finds it well to stretch the perinæum, manually, in advance, so that when the head comes along it will find it pliant and elastic; but, most important of all, he would seek to establish, in the mind of the patient, the great need of avoiding violent expulsive effort while the pressure upon the perinæum is greatest. In this way he thinks much can be done to avoid calamity to the vulvar orifice.

Dr. Keene is not prepared to admit that perineal rents are as frequent as is now generally claimed by the gynecologists, nor that the necessity for their repair, immediate or remote, is as great as would appear by their writings and teachings. Still, in a limited number of cases he would repair the perinæum by immediate suture, as recommended by the essayist. He is a believer in the usefulness of ergot when prudently and timely administered, and thinks the perinæum will generally get along pretty well without much artificial support.

Dr. Lothrop has been in the habit, for the past few years, of treating perineal ruptures by immediate suture, and finds occasion to approve the practice from practical observation of his cases; for, whereas, formerly they were subjected to the slow gettings up and the hundred-and-one annoyances, not to say dangers, of the parturient period, besides the secondary pelvic disorders which unrepaired rents entailed, now the recoveries from the child-bed state are rapid and free from the dangerous and unpleasant conditions, immediate and remote, which the neglected lesion in question is well known to precipitate. As to ergot, he never gave it now-a-days until the termination of the third stage of labor, and he then administered it with a view to obtaining tonic contraction of the muscular structures of the womb, and the consequent shutting up of its sinuses, believing that, thereby, a fruitful avenue of puerperal septicæmia was closed. His practice in regard to perineal support consists rather in pressing the head under the pubic arch and securing flexion, than in any endeavor to give undue support to the perineal body. This period of labor is the critical one, and calls for the exercise of wise judgment and careful management on the part of the accoucheur.

Dr. Banta did not, until some two years ago, even believe that rents of the perinæum following labor were very common, and regarded the lesion, when it did occur, as comparatively unimportant, requiring, at least, no surgical care at the hands of the accoucheur. His practice in this regard was now, however, completely revolutionized. It was almost exceptional when he did not have occasion to suture the perinæum of a primaparous woman. It is so simple and easy to do the operation that he now wonders why he was so long in bringing himself to adopt it, particularly as its benefits are unquestioned. He uses the triangular needle, slightly curved, armed with silk, and invariably obtains good results.

Dr. Potter, in closing the discussion, formulated the salient points of the subject, as follows:

I. As to prevention:

(*a*). Position during delivery: The left-lateral is the best.

(*b*). Refrain from the use of ergot until the completion of the third stage of labor.

(*c*). Protect the perinæum by—

1st. Direct pressure upon the head.

2d. Seizure of the chin through the rectum and enucleation of the head.

3d. The administration of chloroform.

II. In case of a laceration, repair the rent by immediate suture.

Its benefits are—

(*a*). Lessened danger of puerperal septicæmia from absorption of putrescent lochia.

(*b*). The burning pain and irritation of the vulvar orifice during puerperal convalescence are avoided or prevented, in a large measure.

(*c*). Hemorrhage from the torn surfaces is prevented.

(*d*). The patient thereby escapes the danger of future displacements of the pelvic viscera and their long train of distressing phenomena, local and reflex.

(*e*). And finally, the necessity of submitting to the more formidable secondary operation is averted. This is worthy of consideration on economic grounds, even if weightier reasons did not obtain.

